# A randomized trial to compare smooth monofilament suture vs. barbed suture using the three-layer continuous closure technique in canine ovariohysterectomy in a high-quality high-volume spay/neuter clinic

**DOI:** 10.3389/fvets.2024.1365213

**Published:** 2024-04-12

**Authors:** Jacob M. Shivley, W. Cooper Brookshire, Alex P. Shealy, Chase A. Seyer, Philip A. Bushby, Kimberly A. Woodruff

**Affiliations:** Department of Clinical Sciences, College of Veterinary Medicine, Mississippi State University, Mississippi State, MS, United States

**Keywords:** canine ovariohysterectomy, dog spay, suture technique, barbed suture, efficient surgery, high-quality high-volume spay/neuter

## Abstract

The primary objective of this study was to compare time efficiency and complication rates between smooth monofilament suture (SMS) and barbed suture (BS) using the three-layer continuous incisional closure pattern after ovariohysterectomy in a high-quality high-volume spay/neuter clinic. The study was designed as a randomized controlled trial enrolling 71 adult female dogs. Dogs were randomly assigned to SMS or BS treatments. The effect of closure with BS or SMS on closure time was tested through multilevel, multivariable linear regression in a generalized linear mixed model. Body condition score, weight, and pre-closure incision length were tested as covariates. Surgeon was included in the model as a random effect. Pre-closure incision length (*p* = 0.01) and method (*p* ≤ 0.0001) were associated with closure time. Adjusting for pre-closure incision length, the average time for closure with SMS was 6.5 min (range 3.70–10.31 min), and the average time for closure with BS was 4.91 min (range 3.05–8.05 min). Accounting for the closure method, the closure time increased by 39 s for each additional centimeter of incision length. BS was more efficient than SMS when performing the three-layer continuous suture pattern. No short-term telemedicine-assessed complications were noted with either treatment method. BS can improve efficiency in surgical closures, especially considering large volumes of animals, and appears to have a similar short-term, telemedicine-assessed complication rate when compared to SMS.

## 1 Introduction

Surgical efficiency is of great clinical importance and is a topic of interest in medical literature (1–3). Studies concerning human patient outcomes show that surgeons can reduce surgical times and consequently reduce rates of surgical complications and hospital stays (4–8). In fact, when adjusting for patient characteristics, surgeons with longer surgery times had significantly more surgical complications and prolonged patient hospital stays than surgeons with shorter surgery times (9, 10). The veterinary medical literature follows a similar pattern. Duration of surgery can be significantly associated with surgical site infections, and surgical efficiency is often emphasized as one way to reduce postoperative infections (11–13). Shortened surgical times should naturally lead to reduced anesthetic times with a reduction in overall medical costs. A recent retrospective study found that high-volume spay/neuter surgery is associated with very low mortality rates (0.03%), which was attributed to surgical efficiency, proficiency in spay/neuter techniques, and the relatively healthy population of animals presented for surgery (14). High-quality, high-volume spay/neuter (HQHVSN) surgical techniques have been developed and refined to achieve high efficiency, low mortality, and positive outcomes in animals undergoing ovariohysterectomy (OVH) or castration (15–17). Techniques such as the pedicle tie (18), sharp transection of the suspensory ligament (19), and reducing unnecessary ligatures (20) are efficient and safe surgical techniques commonly used in HQHVSN. The encouragement toward efficient surgical wound closure in HQHVSN has been addressed (15, 16, 21), and the innovative three-layer continuous closure technique used in HQHVSN OVH has been suggested as one way to accomplish efficient surgical wound closure (22, 23). The three-layer continuous closure technique is described thoroughly in the Methods section.

The use of barbed suture (BS) has been extensively evaluated in surgery on human patients and has been used in a very wide range of areas including, but not limited to, renal, gastrointestinal, arthrotomy, abdominoplasty, mastopexy, and general incisional closures (24–29). Many of these studies have reported shorter operating times with comparable or decreased complication rates. For example, incisional closure with BS was compared to smooth monofilament suture (SMS) in knee arthroplasty. Closure with BS took approximately 9.8 min on average compared to 14.5 min with SMS with no significant differences in postoperative complications (including surgical site infection) between the groups (30). BS used to close the fascia, subcuticular, and dermal layers after spinal surgery was on average 13 min faster than closure techniques with SMS (8.87 ± 1.721 min for BS vs. 21.85 ± 2.629 min for SMS) with no significant differences in postoperative infection or wound dehiscence (24). Finally, rectus sheath plication and fascial repair using BS in patients undergoing abdominoplasty had on average a 15-min reduction in operative times and eliminated the need for drains (29).

BS has garnered interest among veterinary surgeons due to several reported advantages, including reduced operative time, improved distribution of tension along wound edges, better watertight seals of tissue planes, and reduced tissue strangulation (31–33). Several studies, including laparoscopic gastropexies in dogs, urinary bladder incision closure in goats, oral mucosal wound closures in cats receiving full dental extractions, intradermal skin closure in dogs, and successful tendon repair, have compared closures using SMS vs. BS and reported that BS resulted in shorter incisional closure times (34–38). Regier et al. (31) compared the mechanical properties, strength, and quality of seal for intradermal closures on canine cadavers using SMS vs. BS and concluded that SMS closures were better at withstanding mechanical loads, whereas BS closures provided a superior impermeable seal (31). In an earlier study, Spah et al. reported that laparoscopic gastropexies were performed successfully using BS, and the authors noted that BS allowed for effective intracorporeal laparoscopic suturing of an incisional gastropexy without tying intracorporeal knots (34). There have been fewer reports in the veterinary medical literature on the use of BS in fascial planes or in closing OVH surgical incisions. Bailey et al. evaluated the integrity of the welded-end loop on four different brands of unidirectional BS after transabdominal passage in a canine cadaver (39). They identified one specific brand of BS as having an increased incidence of breaking at the welded-end loop when passed through the body wall, and these findings prevented the authors from recommending this specific type of suture passage through a canine body wall for use in laparoscopic procedures. However, the use of BS in porcine fascial repair showed excellent results in tensile strength and equivalence in postoperative complications when compared to SMS (40). To the author’s knowledge, there is only one report of BS being used to close celiotomies in live-recovery surgeries (41) and there are no reports of it being used to close the abdominal wall, subcutaneous tissue, and dermal tissue in a three-layer continuous manner in live-recovered patients.

The primary objective of this study was to determine whether unidirectional BS was as efficient in performing the three-layer continuous closure as traditional SMS within a group of dogs receiving celiotomy closure, suture choice affected pre-closure vs. post-closure incision length, and perioperative complications were comparable. The authors hypothesized that BS is more efficient than SMS using the three-layer continuous closure pattern in canine OVH and would have equivalent short-term, telemedicine-assessed, complication rates.

## 2 Materials and methods

A randomized controlled clinical trial was designed to test for differences in complication rate and time efficiency between SMS and BS. This study was approved by the Mississippi State University Institutional Animal Care and Use Committee (IACUC-21-278). Signed consent was obtained by shelters and rescue groups prior to animal enrollment.

**Figure 1:**
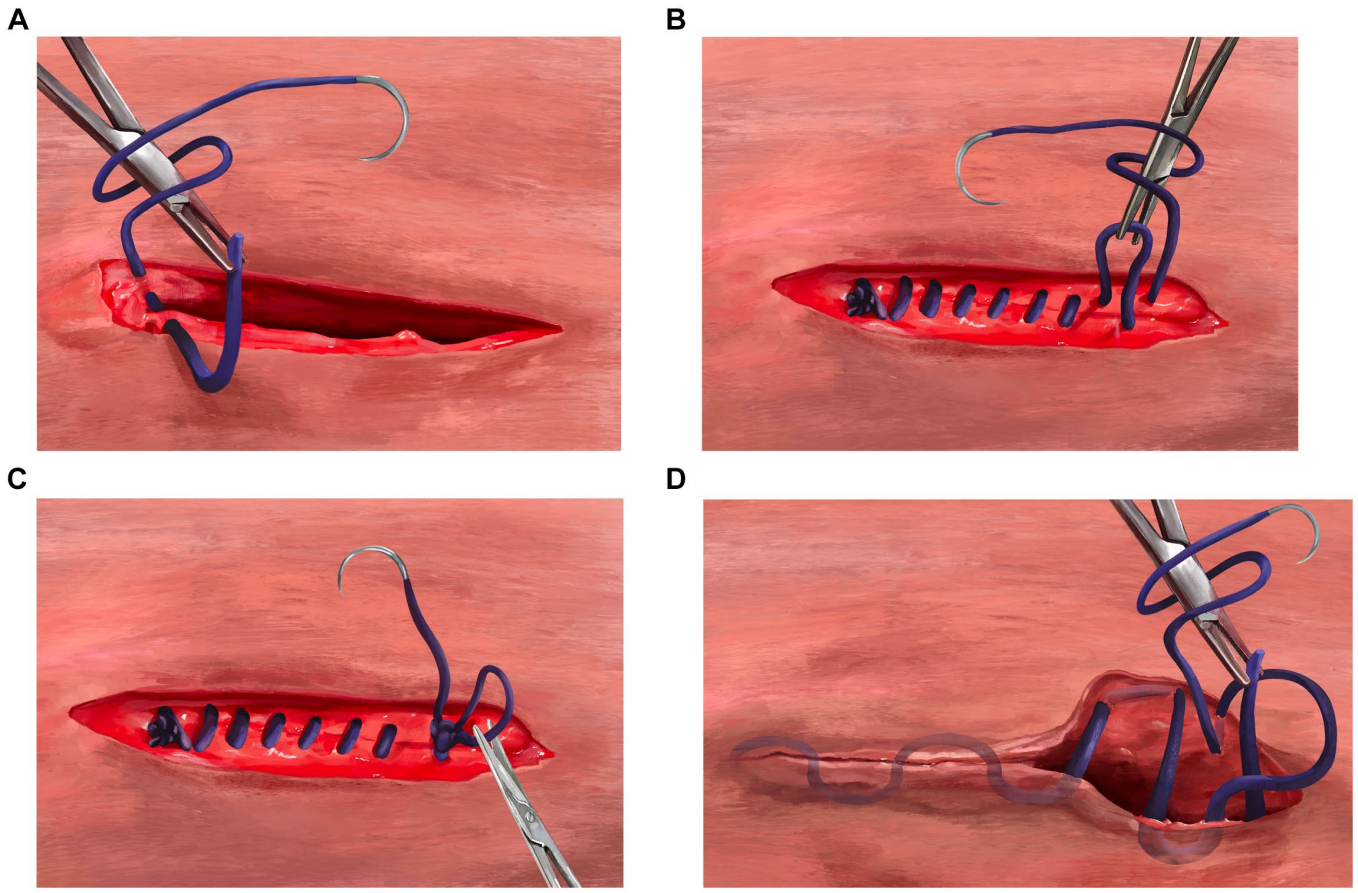
**(A)** Beginning of the body wall closure using the three-layer continuous closure method with smooth monofilament suture. Begin by closing the rectus sheath in a standard simple continuous pattern from the surgeon’s dominant hand toward the non-dominant hand. **(B)** Termination of the body wall closure using the three-layer continuous closure method with smooth monofilament suture. The simple continuous rectus sheath suture line should be terminated with a 6-throw knot using a loop of approximately six centimeters. **(C)** Creating a deep tag for the three-layer continuous closure method using smooth monofilament suture. To properly set up the deep tag, one side of the loop used to terminate the body wall closure is cut near the knot to create a single long tag of suture. The surgeon then would use the long strand with needle to continue directly into a continuous pattern in the subcutaneous tissue occasionally tacking down to the rectus fascia (quilting pattern), moving from the nondominant hand toward the dominant hand. **(D)** Terminating the three-layer continuous closure method after skin apposition using smooth monofilament suture. After the end of the subcutaneous closure, the surgeon should proceed directly into a subcuticular pattern, without tying a knot in the subcutaneous layer. The subcuticular pattern is then performed moving from the dominant hand toward the nondominant hand. At the end of the subcuticular pattern, a “deep strand” is created by taking a bite of dermis in a superficial-to-deep direction. This deep strand is then tied back to the initial tag kept from the external rectus sheath closure with 6 total throws. The suture is trimmed close to the knot to complete the closure.

**Figure 2:**
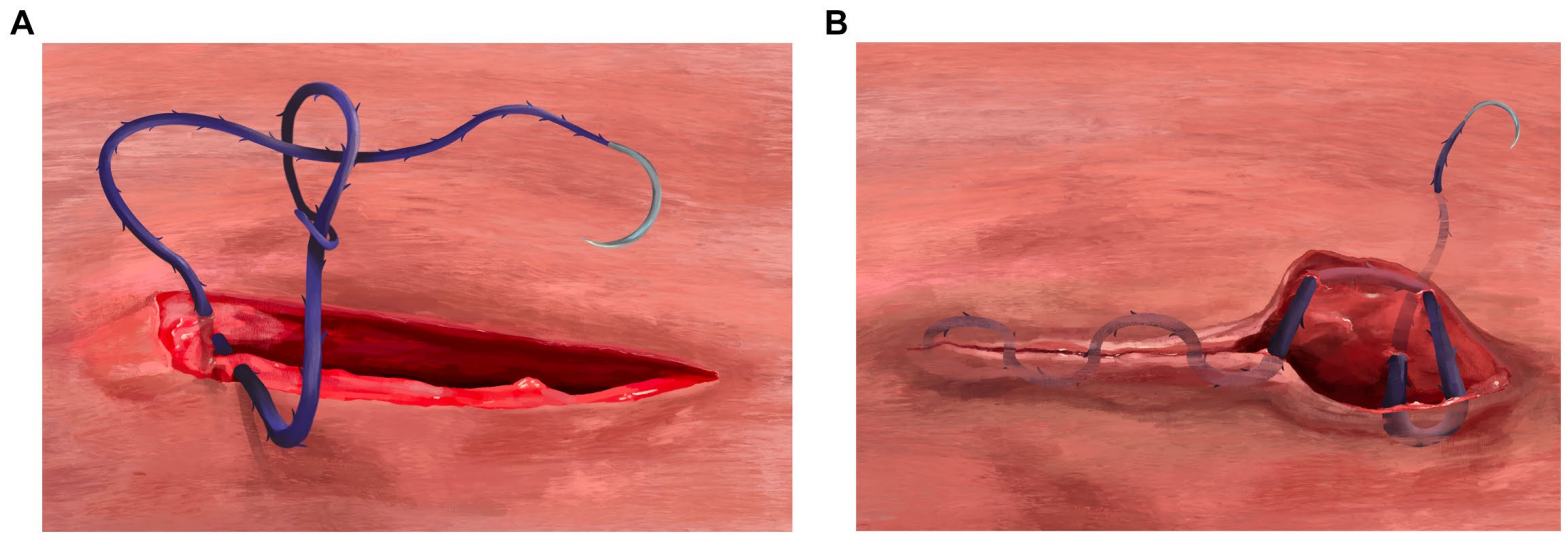
**(A)** Beginning of the body wall closure using the three-layer continuous closure method with barbed suture. After the needle is passed through at least 5 millimeters of external rectus sheath on both sides of the incision, the needle is passed through the variable loop at the end of the suture line and pulled to snugly appose the body wall (no knots are tied). **(B)** Terminating the three-layer continuous closure method after skin apposition using barbed suture. At the very end of the subcuticular pattern using barbed suture, no knots are tied, but the suture must be “anchored” into place. To perform this anchoring technique, a backwards stitch and J stitch is created. The backwards stitch is constructed by placing one final needle pass in the subcuticular tissue moving in the opposite direction (from the non-dominant hand towards the dominant hand). The J stitch is performed in similar fashion as “smurfing” a knot; the needle is passed inside the wound edges deep into the SQ tissue exiting through the epidermis in a perpendicular direction compared to the incision.

The sample population was adult female dogs that were presented for elective OVH at an HQHVSN facility in the southeastern United States between 4 October 2021 and 22 December 2021. To be enrolled in the study, dogs were required to be greater than 6 months old (judged by the presence of fully erupted adult canine teeth) and apparently healthy. Pregnant dogs were excluded from the study. Dogs were randomly assigned, by coin toss, into two groups: A and B. The body wall, subcutaneous tissue, and skin for group A were closed with SMS^1^ in a three-layer continuous fashion, whereas the body wall, subcutaneous tissue, and skin for group B were closed with BS^2^ in a three-layer continuous fashion. The major differences encountered when using BS with a three-layer continuous closure method in comparison with SMS are beginning the rectus sheath closure and terminating the subcuticular pattern at the end of the three-layer closure. Figures 1A–D, 2A,B are provided for comparison.

The size of the suture was pre-determined to remain the same for every dog in each treatment group to reduce variability and bias. Time was measured from the beginning of the closure of the body wall until the suture was cut at the end of the skin closure. The incision length was measured prior to and following closure. Dogs requiring an incision length outside the range of 3–5 cm or additional surgical procedures, such as hernia repair, were excluded.

Five surgeons participated in the study, each with at least 1 year of experience in a high-volume spay/neuter clinic (range = 1–12 years). These surgeons had significant experience with the three-layer continuous closure method using SMS. To familiarize themselves with the BS technique, the surgeons viewed a short multimedia presentation and a surgical training video. Afterward, the surgeons participated in a training session and practiced together using BS on incisions made on practice suture pads prior to the study.

For consistency, one certified veterinary technician (CVT) was designated to record and maintain all data and perform all telemedicine postoperative follow-ups. Surgeons would report the beginning of incisional closure as the beginning of the rectus sheath closure and the ending as the complete termination of the subcuticular pattern at the end of the three-layer closure, so that time could be evaluated with a stopwatch. Surgeons would detail any intra-closure difficulties during this same time frame to the CVT maintaining the stopwatch. Intra-closure difficulties were defined as any issues that interrupted suturing or required the suture line to be restarted (e.g., suture breakage and poor tissue apposition) that occurred during the closure as described above. Surgeons could not be blinded to suture type, but suture type was blinded during statistical analysis.

### 2.1 Treatments

**Figure 3:**
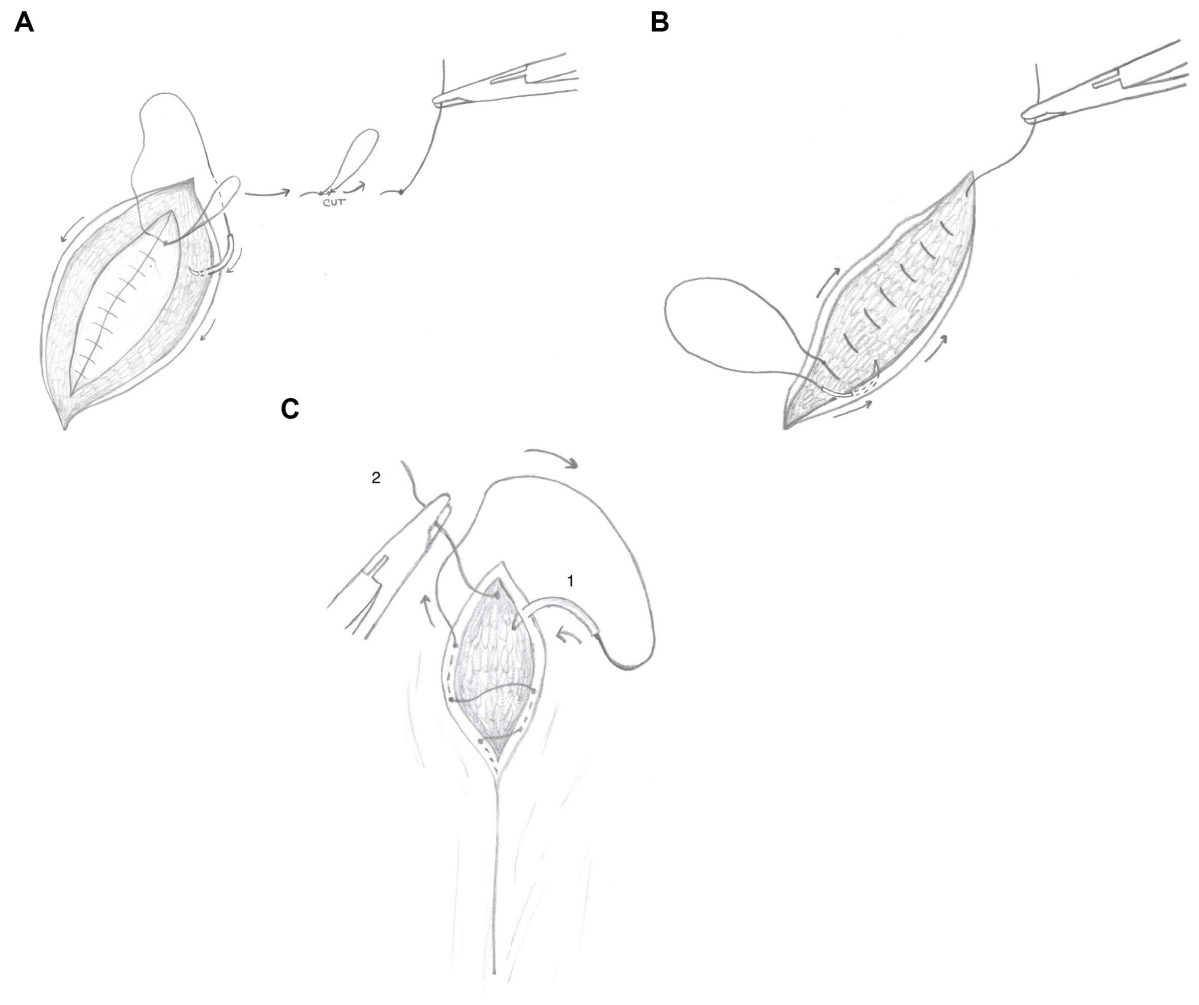
**(A)** Line schematic demonstrating closure using the three-layer continuous closure method with smooth monofilament suture. This line schematic demonstrates the transition from the rectus sheath closure moving into the subcutaneous closure. The dark arrows demonstrate the direction of the subcutaneous closure pattern. **(B)** Line schematic demonstrating closure using the three-layer continuous closure method with smooth monofilament suture. This line schematic demonstrates the transition from the subcutaneous closure moving directly into the subcuticular closure. The dark arrows demonstrate the direction of the subcuticular closure pattern. **(C)** Line schematic demonstrating closure using the three-layer continuous closure method with smooth monofilament suture. The dark arrows denote the directional pattern of the suture. The number 1 designates the final pass of the needle from superficial-to-deep through the dermis. This creates a deep strand to tie to the deep tag (designated by the number 2), thereby burying the final knot.

In group A, the three-layer continuous closure method using SMS (see text footnote 1) was performed by closing the external rectus sheath in a standard simple continuous pattern from the surgeon’s dominant hand toward the non-dominant hand. The simple continuous rectus sheath closure began with a standard six-throw knot and was completed with a six-throw knot using a loop of approximately 6 cm (Figure 3A). One side of the loop was cut near the knot to create a single long tag of suture. The surgeon then moved directly into a continuous pattern in the subcutaneous tissue occasionally tacking down to the rectus fascia [quilting pattern (42)], moving from the non-dominant hand toward the dominant hand. After the end of the incision was reached and the dead space closed, the surgeon proceeded directly into a subcuticular pattern, without tying a knot in the subcutaneous layer (Figure 3B). The subcuticular pattern was then performed moving from the dominant hand toward the non-dominant hand. At the end of the subcuticular pattern, a “deep strand” was created by taking a bite of the dermis in a superficial-to-deep direction (Figure 3C). This deep strand was then tied back to the initial tag kept from the external rectus sheath closure with six total throws. The suture was trimmed close to the knot to complete the closure.

**Figure 4:**
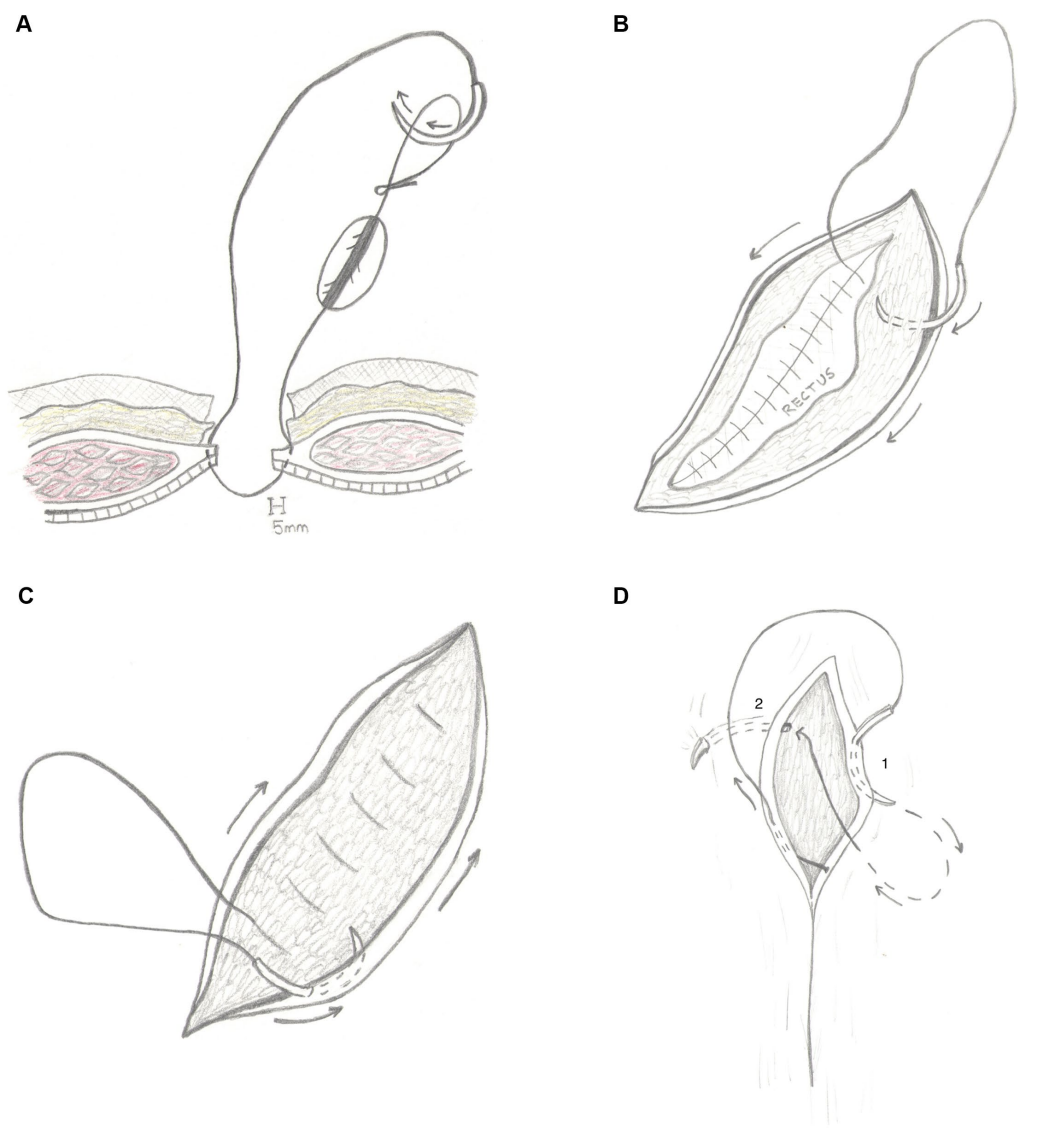
**(A)** Line schematic demonstrating ovariohysterectomy incisional closure using the three-layer continuous technique with barbed suture. After the needle is passed through both sides of the external rectus sheath, the needle is placed through the variable loop at the end of the suture line and pulled to snugly appose the body wall (no knots are tied). **(B)** Line schematic demonstrating ovariohysterectomy incisional closure using the three-layer continuous technique with barbed suture. No knots are tied at the end of the rectus sheath closure. Instead, the surgeon moves directly into the subcutaneous tissue to continue the three-layer continuous pattern, occasionally tacking the tissue to the rectus fascia (quilting pattern). The dark arrows denote the direction of the subcutaneous closure, moving from the nondominant hand towards the dominant hand. **(C)** Line schematic demonstrating ovariohysterectomy incisional closure using the three-layer continuous technique with barbed suture. When the end of the incision is reached and the subcutaneous closure is completed, the surgeon proceeds directly into a subcuticular pattern, without tying a knot in the subcutaneous layer. The dark arrows denote the direction of the subcuticular closure, moving from the dominant hand towards the non-dominant hand. **(D)** Line schematic demonstrating ovariohysterectomy incisional closure using the three-layer continuous technique with barbed suture. The dark arrow depicts the direction of the subcuticular closure pattern. When using barbed suture, at the very end of the subcuticular pattern, the surgeon should not tie a knot. However, the suture must be “anchored” into place. The suture line is terminated by a two-step process. The first step is the backwards stitch, denoted by the number 1. The backwards stitch is constructed by placing one final needle pass in the subcuticular tissue moving in the opposite direction of the rest of the subcuticular pattern. The skin should be apposed after this step. The second step is the J stitch, denoted by the number 2. This is performed in similarly to smurfing a knot; the needle is passed inside the wound edges deep into the SQ tissue exiting through the epidermis in a perpendicular direction compared to the incision. The suture should be cut flush with the skin where the J stitch exits.

In group B, the three-layer continuous closure method using BS (see text footnote 2) was initiated by passing the needle through both sides of the external rectus sheath, then anchored by passing the needle through the variable loop at the end of the suture line and pulled to snugly appose the body wall (no knots were tied; Figure 4A). A continuous suture pattern to close the celiotomy incision was performed, and at the end of the rectus sheath closure, no knots were tied. Instead, the surgeon moved directly into the subcutaneous tissue to create a continuous pattern, occasionally tacking the tissue to the rectus fascia (quilting pattern), moving from the non-dominant hand toward the dominant hand (Figure 4B). After the end of the incision was reached and the dead space closed, the surgeon proceeded directly into a subcuticular pattern, without tying a knot in the subcutaneous layer (Figure 4C). The subcuticular pattern moved in the direction of the dominant hand toward the non-dominant hand. At the very end of the subcuticular pattern, no knots were tied, and instead, the suture was anchored into place using a backward stitch and a J stitch. The backward stitch was created by placing one final needle pass in the subcuticular tissue moving in the opposite direction (from the non-dominant hand toward the dominant hand). The J stitch was created in a similar fashion as “smurfing” a knot; the needle was passed inside the wound edges deep into the SQ tissue exiting through the epidermis in a perpendicular direction compared to the incision (Figure 4D). The backward stitch and J stitch completed the skin closure, and the suture was trimmed where it exited the epidermis.

### 2.2 Pre-surgical measures

Prior to anesthesia, each animal received a physical examination and 5 mg/kg of firocoxib PO. Demographic information (identification, weight, BCS, and estimated age based on dentition) was collected by the designated CVT, and any abnormal findings during physical examination were noted. If significant patient anxiety was present, trazadone at 4–10 mg/kg was administered PO. Each animal was induced for surgery with intramuscular injection (equal amounts of dexmedetomidine (0.5 mg/mL) at 35 mcg/kg, butorphanol (10 mg/mL) at 0.35 mg/kg, and ketamine (100 mg/mL) at 3.5 mg/kg) to provide anesthesia and analgesia. All dogs received eye lubrication and were aseptically prepared for an OVH in a standard fashion.

### 2.3 Surgical procedure

Animals were placed in dorsal recumbency with their front legs secured along the lateral thoracic wall and their rear legs tied to the end of the surgical table. A 4-cm (range 3–5 cm) ventral midline abdominal skin incision was performed just caudal to the umbilicus. Subcutaneous tissue along the linear alba was bluntly or sharply dissected using curved Metzenbaum scissors. A stab incision was made in the linear alba with a #15 scalpel blade, and the incision was extended with scissors. A routine ovariohysterectomy was performed. Prior to incision closure, a sterile stainless steel ruler was used to determine pre-closure incision length by the designated CVT. Incision closure was completed as previously described.

### 2.4 Post-surgical measures

Post-surgical incision length was measured using a stainless steel ruler by the designated CVT. Animals were returned to their respective animal shelter or rescue group after recovering from anesthesia. Dogs were monitored for post-surgical complications, including self-trauma, infection, dehiscence, seroma, and pain at 24, 48 h, and 7 days following surgery by personnel at the animal’s respective animal shelter or rescue group. Each shelter/rescue group agreed to telemedicine monitoring when they signed the client consent form to reduce the chances of exposure to COVID-19. The designated CVT utilized the following postoperative telemedicine script in phone communications at the designated follow-up time: Hello! This is (insert CVT name) calling to follow up on the animals in your care who received surgery with the shelter program. I wanted to specifically ask about (insert names of dogs).

1. Have you noticed (insert name of dog) licking or chewing at the incision?
2. Have you seen any swelling or fluid accumulation under or around the incision?
3. Is the incision still sealed together?
4. Please send a picture of the incision to xx@xx.edu.
  - Please make it very clear which dog is in the picture.

Shelter and rescue group personnel were instructed to contact the CVT if any complications or issues were encountered after the 7-day follow-up protocol. In addition to the complication reporting by shelters and rescue groups, long-term follow-up calls were made by the designated CVT approximately 6 months after each animal’s surgery to assess whether any major complications occurred after the short-term follow-up protocol.

### 2.5 Statistical analysis

Prior to commencing the study, a sample size estimate was calculated using pilot data previously collected. These pilot data suggested that closure time for BS and SMS would be approximately 5 and 6 min, respectively. The estimated standard deviation in time was set at 75 s. The planned sample size of 35 surgeries in each treatment group was determined to be sufficient for descriptive statistics and comparisons between closure methods. Assuming α = 0.05 and utilizing previously collected pilot data, 35 surgeries in each group provided 90% power to detect a clinical and statistical difference in closure time between methods. The data were tested for homoscedasticity. All data were found to be normally distributed. The effect of closure with BS or SMS on closure time was tested through multilevel, multivariable linear regression in a generalized linear mixed model.^3^ Body condition score, weight, and pre-closure incision length were tested as covariates. Surgeon was included in the model as a random effect. An interaction between the surgeon and the method was tested by using the surgeon as a fixed effect. The effect of the closure method on the change between pre-closure and post-closure incision length was measured for 69 dogs and tested through multilevel, multivariable linear regression in a generalized linear mixed model (see text footnote 3). Body condition score, weight, and method were tested as covariates. Surgeon was included as a random effect. Both models were built using manual forward variable selection; variables were retained in the model if the type III *p*-value was statistically significant, and inclusion of the variable improved model fit using the Akaike information criterion (AIC). Significance was set *a priori* at alpha = 0.05.

## 3 Results

In total, 71 dogs were included in the study (SMS, *n* = 35; BS, *n* = 36). The average weight of the dogs included in the study was 16.7 kg (median = 16.5, range = 3–28.5), with an average body condition score (scale of 1–9) of 5 (median 5, range 3–8). The average age of the sample population was 3.4 years (median = 3, ranges = 0.5–7). One dog (SMS) experienced an intra-closure difficulty (broken suture). This patient was not excluded from the study and was included in the statistical analyses. No intra-operative, short-term, or long-term telemedicine-assessed postoperative complications were reported for either closure method. No animals were lost to follow-up during the short-term period, but 16 animals were lost to follow-up at the 6-month assessment period. The information during the long-term assessment period was received for 49 animals, with an even distribution between the CC (*n* = 25) and BS (*n* = 24) groups.

The average weight of the dogs that were closed with BS was 15.4 kg (median = 15.8, range = 3–27.2) with a body condition score of 5 (median = 5, range = 3–8). The average age was 3.17 years (median = 3, range = 0.5–7). The average weight of the dogs that were closed with SMS was 17.9 kg (median = 17.5, ranges = 3.5–28.5) with an average body condition score of 4.8 (median = 5, ranges = 3–8). The average age for dogs closed with SMS was 3.4 years (median = 4, ranges = 0.5–7). The average pre-closure incision length for SMS was 3.89 cm (range = 3–4.5 cm) and 4.02 cm (range = 3–5 cm) for BS. The average post-closure incision length was 3.8 cm (range = 3–4.5 cm) for monofilament and 3.9 cm (range = 2.5–5 cm) for BS. The data met the assumptions of homoscedasticity and normality of residuals. Two dogs (1 SMS, 1 BS) had incomplete data related to the incision length and were excluded from statistical analyses involving incision length. There were no associations found between weight, body condition score, age, or closure method and the difference between pre-closure and post-closure length.

Four dogs (1 SMS, 3 BS) had incomplete data related to closure time and were excluded from statistical analyses involving closure time. The method of closure was associated with closure time (*p* ≤ 0.0001). Surgeries performed with BS had a pre-closure incision length adjusted time of 4.91 min (range 3.05–8.05 min), while surgeries performed with SMS had a pre-closure incision length adjusted time of 6.5 min (range 3.70–10.31 min). On average, surgeries performed with BS had an adjusted time of 1.59 fewer minutes, compared to SMS. Pre-closure incision length (*p* = 0.01) was also associated with closure time. Accounting for the closure method, the closure time increased by 39 s for each additional centimeter of incision length. There was no interaction between the surgeon and method (*p* = 0.4) and no interaction between the method and incision length (*p* = 0.6). There was no association between suture type and pre-closure vs. post-closure incision length.

## 4 Discussion

These data demonstrate both a clinically and statistically significant difference in surgical times between SMS and BS when performing the three-layer continuous OVH closure. When compared to SMS, the use of BS with the three-layer continuous technique reduced closure time by approximately 1.59 min, when adjusting for incision length. For such small incisions, time efficiency may be clinically negligible for individual patients. However, when considering longer incisions or when compared to traditional closure methods, time efficiency could be clinically significant for individual patients when using BS. Additionally, surgical time reduction results in veterinarian and veterinary support staff labor savings. For example, in an HQHVSN clinic that performs 20 dog spays per day, the time savings seen in this study would add up to 32 min over the course of a day leading to over 130 h over the course of a year.

Some studies suggest that while unidirectional BS may improve watertight skin closure, surgeons should consider using conventional monofilament sutures when the mechanical strength of the closure is of primary concern (31, 38). These studies examined sutures with different absorption profiles than the sutures used in our study (PDO), so direct clinical comparisons are difficult. However, based on observations in our study in a high-volume context, it would appear that BS with PDO absorption profiles can withstand the mechanical stress placed on body wall closures. No shelters or rescue groups for any patient enrolled in this study reported significant surgical complications during or past the initial 7-day postoperative period. There were also no complications reported during the 6-month long-term follow-up assessment. Approximately 25% of individuals in the study were lost to follow-up during the 6-month assessment, so it is possible that undetected complications occurred in these patients.

An article reporting the successful repair of a complete common calcanean tendon rupture identified a potential difficulty when using BS; once the barbs lock into the tissue, the suture is unable to be backed out (37). This could be an issue with novice surgeons or those who have not developed the skill from deliberate practice with exact suture placement in tissues. We did not experience any intra-closure difficulties with BS. The only intra-closure difficulty encountered was during one surgery with SMS; the suture broke during the first-knot placement in the body wall, and the closure had to be restarted.

Potential weaknesses of this study include enrollment limited to apparently healthy dogs presenting for OVH at an HQHVSN clinic, a small number of surgeons, lack of surgeon blinding, specification differences between BS and SMS, and a small sample size. Bias may have been introduced by enrolling only five surgeons who had minimal experience with BS to participate in this study, but the surgeon effect was measured and accounted for in our statistical model. The needle and suture specifications of the smooth monofilament and BS were not the same. The BS was a larger gauge as recommended by the manufacturer. The manufacturer recommends a one-step increase in the suture gauge for equivalent tensile strength (e.g., 0 BS has equivalent tensile strength to 2–0 SMS). This study was limited to a sample size of 71 dogs, which was sufficient to demonstrate a statistically and clinically significant difference in surgical time between the groups. However, this sample size was not sufficient to evaluate potential differences in short-term postoperative complications, as no dogs in the study experienced a complication during the short-term perioperative phase. Despite the lack of power in our statistical analysis of complication rates, our findings suggest that there is a low risk of telemedicine-assessed postoperative complications for BS in the short-term postoperative phase (7 days) and based on no reported complications beyond 7 days. Due to the nature of high-volume spay/neuter clinics and the fact that this study was performed during the COVID-19 pandemic, shelter and rescue group personnel performed the assessments of potential complications. Although severe complications (dehiscence, SSI, and large seromas) would likely be observed, mild issues (redness, mild swelling, and minor discharge) may be missed during the observational period. The long-term, 6-month assessment information received for 49 animals corroborates the lack of major complications but is still subject to the limitations previously mentioned. In future studies, direct veterinarian evaluation should be considered during the postoperative observation phase in patients and this phase could last 30 days.

The use of SMS to create the three-layer continuous closure pattern when closing adult OVH incisions in dogs has not been extensively described in the veterinary literature (22). To the authors’ knowledge, this closure pattern has not been examined in a prospective study for efficiency and complication rate, nor has it been compared to other closure methods. This method has been adapted from previously described HQHVN closure techniques (21, 43) and has been routinely used by the authors for the past 4 years and anecdotally has seen much success in creating secure OVH closures. To reduce bias and increase reliability, we chose to use this method as the closure method, as the technique using BS was strikingly similar to the three-layer continuous pattern. Future studies comparing the three-layer continuous closure method to traditional closure methods are warranted.

We hypothesize that our findings of increased efficiency and no difference in short-term peri-operative complications when using BS in the three-layer continuous pattern in OVH incision closures are generalizable to other types of abdominal surgeries in dogs. Additional studies are needed to test this hypothesis. Furthermore, it is recommended for future studies to evaluate long-term follow-up to address mechanical strength and wound healing before these findings can be generalized to all dog populations. Future studies could also include dogs of all ages, other species such as cats, and other types of surgical wounds involving fascial planes.

## 5 Conclusion

In conclusion, the results of this study support our hypothesis that BS is more efficient than SMS using the three-layer continuous closure pattern in canine OVH and has equivalent short-term complication rates in the context of the HQHVSN environment.

## Data availability statement

The raw data supporting the conclusions of this article will be made available by the authors, without undue reservation.

## Ethics statement

The animal study was approved by Mississippi State University Institutional Animal Care and Use Committee (IACUC-21-278). The study was conducted in accordance with the local legislation and institutional requirements.

## Author contributions

JS: Conceptualization, Data curation, Investigation, Methodology, Project administration, Resources, Supervision, Writing – original draft, Writing – review & editing. WB: Conceptualization, Data curation, Formal analysis, Investigation, Methodology, Writing – original draft, Writing – review & editing. AS: Investigation, Writing – original draft, Writing – review & editing. CS: Investigation, Writing – original draft, Writing - review & editing. PB: Conceptualization, Investigation, Writing – original draft, Writing – review & editing. KW: Conceptualization, Data curation, Formal Analysis, Investigation, Methodology, Writing – original draft, Writing – review & editing.

## Footnotes

^1^2-0 PDS II; CT-1 36 mm 1/2c Taper. Ethicon, Inc., US, LLC, Somerville, NJ, USA.

^2^0 Quill® PDO; CP-1 36 mm 1/2c Reverse Cutting. Corza Medical, Westwood, MA, USA.

^3^PROC MIXED, SAS, for Windows v9.4, SAS Institute, Inc., Cary, N.C, USA.
